# Triglyceride-glucose-waist circumference index predicts the incidence of cardiovascular disease in Korean populations: competing risk analysis of an 18-year prospective study

**DOI:** 10.1186/s40001-024-01820-9

**Published:** 2024-04-02

**Authors:** Sung Ho Ahn, Hye Sun Lee, Jun-Hyuk Lee

**Affiliations:** 1 Department of Family Medicine, Gangnam Severance Hospital, Yonsei University College of Medicine, Seoul, 03722 Republic of Korea; 2 Biostatistics Collaboration Unit, Department of Research Affairs, Yonsei University College of Medicine, Seoul, 03277 Republic of Korea; 3 Department of Family Medicine, Nowon Eulji Medical Center, Eulji University School of Medicine, Seoul, 01830 Republic of Korea

**Keywords:** Cardiovascular disease, Triglyceride and glucose-waist circumference index, Insulin resistance, Mortality, Cohort

## Abstract

**Background:**

The triglyceride and glucose-waist circumference (TyG-WC) index demonstrated a strong association with insulin resistance, especially in Asian population. However, evidence on the association between TyG-WC index and the occurrence of cardiovascular disease (CVD) is limited. This study aimed to verify association between the TyG-WC index and the occurrence of CVD by considering all-cause mortality as a competing risk.

**Methods:**

The study included 7482 participants divided into four groups based on the TyG-WC index quartiles. Kaplan–Meier curves illustrated cumulative incidence rates of CVD and all-cause mortality during the follow-up period. Log-rank tests determined group differences. The Cox proportional hazard spline curve demonstrates the dose-dependent relationship between the TyG-WC index and incident CVD. Modified Cox regression (Fine and Gray) estimated hazard ratios (HRs) with 95% CIs for incident CVD, treating death as a competing risk. Death event after incident CVD was excluded from the death count.

**Results:**

During the median 15.94 year of follow-up period, a total of 691 (9.24%) new-onset CVD cases and 562 (7.51%) all-cause mortality cases were confirmed. Cox proportional hazard spline curves suggested that TyG-WC index exhibited a dose-dependent positive correlation with incident CVD. The cumulative incidence rate of CVD was significantly higher in the groups with higher TyG-WC index quartiles in Kaplan–Meier curves. The adjusted HR (95% CI) for incident CVD in Q2–Q4, compared with Q1, was 1.47 (1.12–1.93), 1.91 (1.44–2.54) and 2.24 (1.63–3.07), respectively. There was no significant association between TyG-WC index and all-cause mortality. Specifically, angina and stroke were significantly associated with the TyG-WC index, in contrast to myocardial infarction and peripheral artery disease.

**Conclusions:**

The TyG-WC index was positively associated with incident CVD even considering all-cause mortality as a competing risk. Therefore, TyG-WC index may be a valuable marker for predicting the occurrence of CVD.

**Supplementary Information:**

The online version contains supplementary material available at 10.1186/s40001-024-01820-9.

## Introduction

Cardiovascular disease (CVD) stands as the foremost contributor to global morbidity and mortality [1, 2]. In 2020**,** CVD was responsible for approximately 19 million deaths worldwide and attributed to nearly 31% of all deaths in 2017 [3, 4]. Insulin resistance (IR) is a well-known predictor of CVD and mortality [5, 6]. The hyperinsulinemic-euglycemic clamp test is the gold standard for assessing IR [7, 8]. However, its invasiveness and complexity have led to the use of indirect clinical methods, such as the homeostatic assessment of insulin resistance (HOMA-IR) and triglyceride glucose (TyG) index [9, 10]. In particular, there is growing evidence that the TyG index remains superior as compared to the HOMA-IR [11–13]. More recently, further evidence suggests that TyG index-related markers, such as TyG-waist circumference (WC) and TyG-body mass (BMI) indices, demonstrated a stronger association with IR than the TyG index alone [14, 15]. In Asian populations, the TyG-WC index exhibits the highest explanatory power for IR among the TyG index and its related markers [16, 17].

There is firm evidence of an association between the TyG index and the occurrence of CVD [18–20]. Several studies have also indicated a positive correlation between TyG-related markers and cardio-metabolic disorders, including myocardial infarction and coronary artery calcification, among individuals at high risk for CVD [21–23]. However, evidence regarding the relationship between the TyG-WC index and CVD incidence among the general population remains limited. Moreover, given the increased mortality risk in individuals with IR [24, 25], it remains crucial to consider mortality as a potential competing risk when associating the TyG-WC index and CVD incidence. Thus, incorporating mortality as a competing risk in the statistical analyses can enhance the validity of study findings.

From this perspective, this study aimed to explore the potential association between the TyG-WC index and the occurrence of CVD by considering all-cause mortality as a competing risk and by analyzing data from a community-based prospective cohort.

## Materials and methods

### Study population

The data used in this study were obtained from the Korean Genome and Epidemiology Study (KoGES) Ansan-Ansung cohort. The KoGES Ansan-Ansung cohort was a longitudinal, community-based study conducted biennially by the Korea Disease Control and Prevention Agency (KDCA), starting from the baseline survey in 2001–2002 and continuing until the ninth follow-up in 2019–2020. This cohort was comprised of 10,030 participants aged 40–69 years. The participants were divided into two groups: 5018 urban inhabitants from Ansan and 5012 rural inhabitants from Ansung; all of whom had resided in their respective areas for at least 6 months. At each visit, detailed information regarding personal medical histories, anthropometric measurements, and blood sample data were collected from each participant.

**Fig. 1:**
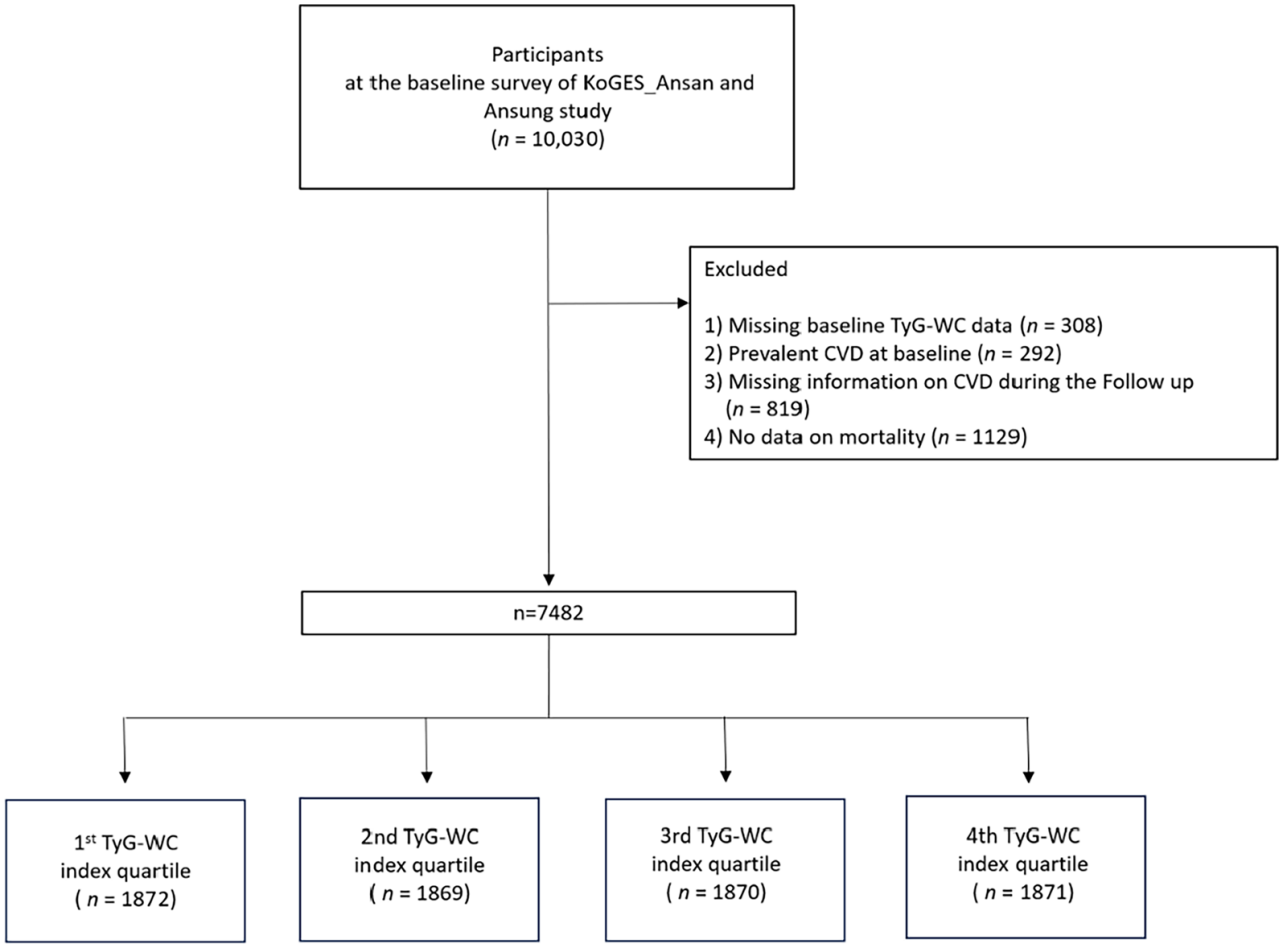
Flowchart of the study population

The participants were tracked from the date of the baseline survey until the date of the first confirmed CVD event, study endpoint, or last contact. The follow-up period was defined as the time between the baseline visit and the occurrence of new-onset CVD events. Figure 1 presents an overview of the study population selection process. We applied the following exclusion criteria: (1) 308 individuals for whom the TyG-WC index could not be calculated due to missing TyG index and/or WC data; (2) 292 individuals with a history of prevalent CVD at baseline; (3) 819 participants without follow-up CVD data; and (4) 1129 individuals without available mortality information. Ultimately, 7482 participants were included in the study and divided into four groups based on TyG-WC index quartiles. These groups consisted of the lowest (Q1, *n* = 1872), second (Q2, *n* = 1869), third (Q3, *n* = 1870, and the highest quartiles (Q4, *n* = 1871), respectively.

The KoGES Ansan-Ansung cohort protocol was approved by the Institutional Review Board (IRB) of the KDCA. All participants provided written informed consent. The study protocol adhered to the ethical principles outlined in the 1964 Declaration of Helsinki and its subsequent revisions. This study was approved by the IRB of Nowon Eulji Medical Center (IRB number: 2022-12-010).

### Data collection

Each participant’s height and weight were meticulously measured to the nearest 0.1 cm and 0.1 kg, respectively. Subsequently, BMI was calculated in kilograms per square meter (kg/m^2^) and obesity was defined as BMI ≥ 25 kg/m^2^ [26]. WC measurements were conducted with precision and recorded to the nearest 0.1 cm in a horizontal plane situated midway between the lowest rib and the iliac crest. Blood pressure measurements included both systolic and diastolic blood pressures, which were recorded as the average of the last two measurements; the mean blood pressure was subsequently derived from these values. After a minimum fasting period of 8 h, blood samples were collected from each participant for further analysis. These samples were then subjected to laboratory analysis for assessing key parameters, including fasting plasma glucose, serum insulin, total cholesterol, triglycerides, high-density lipoprotein cholesterol, and C-reactive protein (CRP) levels. In cases with serum triglyceride levels < 400 mg/dL, serum low-density lipoprotein cholesterol levels were calculated using the Friedewald formula. The estimated glomerular filtration rate (eGFR) was calculated using the CKD Epidemiology Collaboration (CKD-EPI) equation [27].

Hypertension (HTN) was defined as meeting one of the following criteria: (1) systolic blood pressure ≥ 140 mmHg; (2) diastolic blood pressure ≥ 90 mmHg; or (3) receiving treatment with anti-hypertensive medications [28]. Diabetes mellitus (DM) was defined as meeting one of the following criteria: (1) fasting plasma glucose level ≥ 126 mg/dL; (2) plasma glucose level ≥ 200 mg/dL at 2 h after a 75-g oral glucose tolerance test; (3) glycosylated hemoglobin level ≥ 6.5%, or receiving treatment with; (4) anti-diabetic medications; or (5) insulin therapy [29]. Dyslipidemia was defined as meeting one of the following criteria: (1) serum total cholesterol concentration ≥ 240 mg/dL; (2) low-density lipoprotein cholesterol concentration ≥ 160 mg/dL; (3) high-density lipoprotein cholesterol concentration < 40 mg/dL; (4) triglyceride concentration ≥ 200 mg/dL; or (5) receiving treatment with lipid-lowering medications [30].

The participants were also asked to complete self-reported questionnaires that comprehensively covered their dietary habits, smoking status, alcohol consumption, and physical activity. Dietary assessments were conducted using a rigorously validated, 103-item, semi-quantitative food frequency questionnaire, thereby enabling the computation of total daily energy intake (kcal/day). Smoking habits were categorized as “never smoked”, “ex-smoker”, “intermittent smoker”, or “daily smoker”. Alcohol intake (g/day) was calculated by multiplying the average pure alcohol content (10 g per glass of alcoholic beverage) with the number of glasses consumed per occasion and the frequency of alcohol consumption (times per day). Participants were classified as either “current drinkers” or “non-drinkers”. Physical activity levels were assessed using the International Physical Activity Questionnaire; the metabolic equivalent of task hours per day (MET-h/day) was also estimated. Based on these values, participants were categorized into three groups reflecting their physical activity levels: (1) low (less than 7.5 MET-hr/day); (2) moderate (ranging from 7.5 to 30 MET-hr/day); and (3) high (exceeding 30 MET-hr/day). Educational level was categorized into three groups: (1) elementary or middle school; (2) high school; or (3) college/university. Monthly household income was categorized into three groups: (1) < 1 million Korean Won (KRW); (2) 1–2 million KRW; and (3) > 2 million KRW.

### Assessment of TyG-WC index

The TyG and TyG-WC indices were calculated using the following formula [21–23]:

$${\text{TyG index}}\, = \,{\text{ln }}[{\text{fasting serum triglyceride }}\left( {{\text{mg}}/{\text{dL}}} \right)\, \times \,{\text{fasting plasma glucose }}\left( {{\text{mg}}/{\text{dL}}} \right)/{2}]$$

$${\text{TyG}} - {\text{WC index}}\, = \,{\text{TyG index}}\, \times \,{\text{WC }}({\text{cm}})$$

### Assessment of CVD

Incident CVD was defined as a newly developed myocardial infarction, angina pectoris, peripheral artery disease, or stroke. When a participant reported the occurrence of a CVD event via personal medical history questionnaires, well-trained examiners conducted comprehensive personal interviews for appropriate confirmation. To gather data on all-cause mortality, this study linked the unique personal identification key code generated by the KoGES with national data sources, thereby specifically obtaining the death records from the Korea National Statistical Office. This process involved tracking information related to the cause and date of death of the participants from January 2001 to December 2020. The underlying causes of death were determined based on the Korean Standard Classification of Disease codes listed in the National Death Index. In instances where multiple subtype events occurred within the same period, they were aggregated as a single CVD event. Consequently, the total number of incident CVD events amounted to 691, alongside a total of 771 subtype events.

### Statistical analysis

Continuous variables are expressed as mean ± standard deviation or median (25th, 75th); categorical variables are expressed as numbers (percentage, %). To investigate the clinical characteristics of our study population, we used one-way analysis of variance or Kruskal–Wallis H test for continuous variables and chi-square tests for categorical variables to assess the differences among the TyG-WC index quartile groups. The Cox proportional hazard spline curve was employed to demonstrate the dose-dependent relationship between the TyG-WC index and incident CVD. The cumulative incidence rates of CVD and all-cause mortality during the follow-up period were depicted using Kaplan–Meier curves. Log-rank tests were used to determine the distribution of the cumulative incidence rate of CVD and all-cause mortality among the groups. Assumptions of proportional hazards were verified through log–log plots; no deviations were observed (Additional file 1: Fig. S1). As the influence of competing risks in prognostic model development has become increasingly recognized [31], we utilized the modified Cox regression approach developed by Fine and Gray to estimate the hazard ratios (HRs) and 95% CI of CVD incidence. In this analysis, we considered death as a competing risk and excluded mortality cases if an incident CVD event occurred prior to death, which was the case in 71 instances. In the multivariate model, age, sex, BMI, smoking status, drinking status, residence, physical activity, total energy intake, mean blood pressure, fasting plasma glucose, serum total cholesterol, CRP and eGFR were included. Each of the four subtypes of CVD was further analyzed to explore its association with the TyG-WC index. Additionally, subgroup analyses stratified by age, sex, obesity, HTN, DM and dyslipidemia were performed. Sex, age, body mass index, mean blood pressure, fasting plasma glucose, and serum total cholesterol levels were excluded from the subgroup analysis for each component. All statistical analyses were conducted using the R software (version 4.1.1; R Foundation for Statistical Computing, Vienna, Austria). The significance level was set at *P* value < 0.05.

## Results

### General characteristics of the study population

Table 1 presents the clinical characteristics of the 7482 participants categorized into quartiles based on the TyG-WC index. The lowest proportions of men and rural inhabitants, along with the youngest mean age, were observed in Q1, whereas Q4 exhibited the oldest mean age and the highest proportions of men and rural inhabitants. The mean eGFR was highest in Q1, with Q4, Q3 and Q2 following in descending order. As the TyG-WC index quartiles increased, there was a noticeable increase in mean WC, BMI, mean blood pressure, fasting plasma glucose, and serum triglyceride levels. Furthermore, there was a progressive increase in the proportion of current drinkers and smokers as the TyG-WC index quartiles increased, whereas the percentage of never-smokers decreased. Average daily energy intake increased with higher TyG-WC index quartiles. Furthermore, there was a positive association between serum CRP levels and TyG-WC index quartiles.

**Table 1:** Baseline characteristics of the study population

Variables	Overall (*n* = 7482)	TyG-WC index quartiles				*P value
		Q1	Q2	Q3	Q4	
		(*n* = 1872)	(*n* = 1869)	(*n* = 1870)	(*n* = 1871)	
TyG-WC index	718.9 ± 101.8	592.5 ± 37.5	680.1 ± 20.5	750.8 ± 20.7	852.2 ± 54.0	< 0.001
Male sex, n (%)	3515 (47.0%)	596 (31.8%)	890 (47.6%)	986 (52.7%)	1043 (55.7%)	< 0.001
Age, years	51.6 ± 8.6	49.3 ± 8.4	51.6 ± 8.6	52.3 ± 8.4	53.3 ± 8.5	< 0.001
BMI, kg/m^2^	24.6 ± 3	21.8 ± 2.2	23.8 ± 2.2	25.3 ± 2.2	27.3 ± 2.7	< 0.001
MBP, mmHg	96.3 ± 13.1	90.1 ± 12.0	95.0 ± 12.3	98.0 ± 12.2	102.0 ± 12.7	< 0.001
Waist circumference	82.5 ± 8.7	72.0 ± 4.4	79.9 ± 3.6	85.6 ± 3.9	92.4 ± 5.7	< 0.001
Smoking status, n (%)						< 0.001
Never smoker	4563 (61.8%)	1365 (74.1%)	1141 (61.4%)	1056 (57.5%)	1001 (54.0%)	
Ex-smoker	1112 (15.1%)	150 (8.1%)	286 (15.4%)	318 (17.3%)	358 (19.3%)	
Intermittent smoker	159 (2.2%)	27 (1.5%)	48 (2.6%)	43 (2.3%)	41 (2.2%)	
Daily smoker	1554 (21.0%)	300 (16.3%)	382 (20.6%)	419 (22.8%)	453 (24.4%)	
Current drinker, n (%)	3561 (48.0%)	774 (41.7%)	899 (48.4%)	931 (50.4%)	957 (51.6%)	< 0.001
Physical activity, n (%)						< 0.001
Low	542 (7.5%)	133 (7.4%)	127 (7.0%)	131 (7.4%)	151 (8.4%)	
Moderate	4397 (61.1%)	1182 (65.6%)	1107 (61.2%)	1047 (58.8%)	1061 (59.0%)	
High	2253 (31.3%)	488 (27.1%)	576 (31.8%)	602 (33.8%)	587 (32.6%)	
Monthly household income, n (%)						< 0.001
< 1 million Korean won	2439 (33.2%)	490 (26.6%)	598 (32.6%)	628 (34.3%)	723 (39.3%)	
1–2 million Korean won	2179 (29.7%)	592 (32.1%)	529 (28.9%)	557 (30.5%)	501 (27.3%)	
> 2 million Korean won	2726 (37.1%)	762 (41.3%)	706 (38.5%)	644 (35.2%)	614 (33.4%)	
Education level, n (%)						< 0.001
Elementary/middle school	4065 (54.8%)	899 (48.4%)	1019 (55.0%)	1049 (56.6%)	1098 (59.2%)	
High school	2343 (31.6%)	705 (37.9%)	602 (32.5%)	547 (29.5%)	489 (26.4%)	
College/University	1012 (13.6%)	255 (13.7%)	231 (12.5%)	258 (13.9%)	268 (14.4%)	
Residence, n (%)						< 0.001
Rural area	3688(49.3%)	725(38.7%)	885(47.4%)	994(53.2%)	1084(57.9%)	
Urban area	3794(50.7%)	1147(61.3%)	984(52.6%)	876(46.8%)	787(42.1%)	
Energy intake, kcal/day	1969.9 ± 715.1	1915.0 ± 692.3	1946.5 ± 678.1	1987.0 ± 733.0	2031.5 ± 750.1	< 0.001
FPG, mg/dL	86.8 ± 20.3	80.5 ± 8.9	83.7 ± 12.3	86.5 ± 17.2	96.6 ± 31.2	< 0.001
Total cholesterol, mg/dL	184.4 ± 140.6	183.3 ± 138.7	180.9 ± 134.9	184.6 ± 138.2	188.8 ± 150.1	0.390
Triglyceride, mg/dL	159.9 ± 100.8	99.2 ± 32.3	128.1 ± 46.3	165.1 ± 68.2	247.3 ± 143.1	< 0.001
eGFR, mL/min/1.73m^2^	92.7 ± 13.8	96.0 ± 13.4	91.5 ± 13.5	90.0 ± 14.1	92.7 ± 13.8	< 0.001
CRP, mg/dL	0.14 [0.06;0.24]	0.1 [0.03;0.18]	0.14 [0.06;0.22]	0.14 [0.07;0.25]	0.18 [0.1;0.31]	< 0.001

BMI, body mass index; CRP, C-reactive protein; eGFR, estimated glomerular filtration rate; FPG, fasting plasma glucose; MBP, mean blood pressure

^*^P value for the comparison of the baseline characteristics among four different TyG-WC index quartile groups. For continuous variables, except for CRP, one-way analysis of variance was used. The Kruskal–Wallis H test was used to compare serum CRP levels among the groups. For categorical variables, chi-square test was used. Significance was set at *P* < 0.05

### Association between TyG-WC index and CVD considering all- cause death as competing risk

**Fig. 2:**
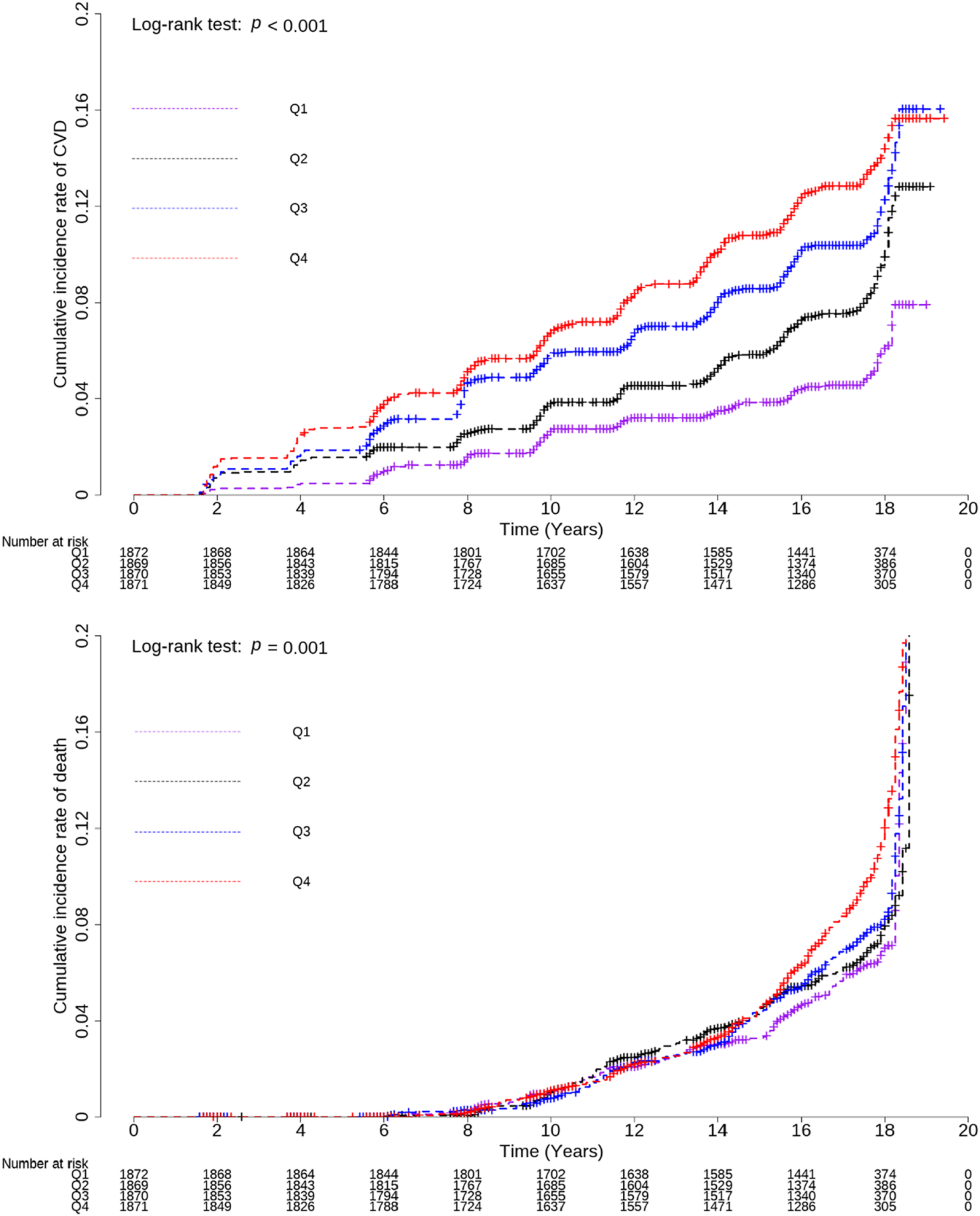
Kaplan–Meier curves showing the cumulative incidence rate of CVD event and all-cause mortality. Abbreviations: CVD, cardiovascular disease

**Fig. 3:**
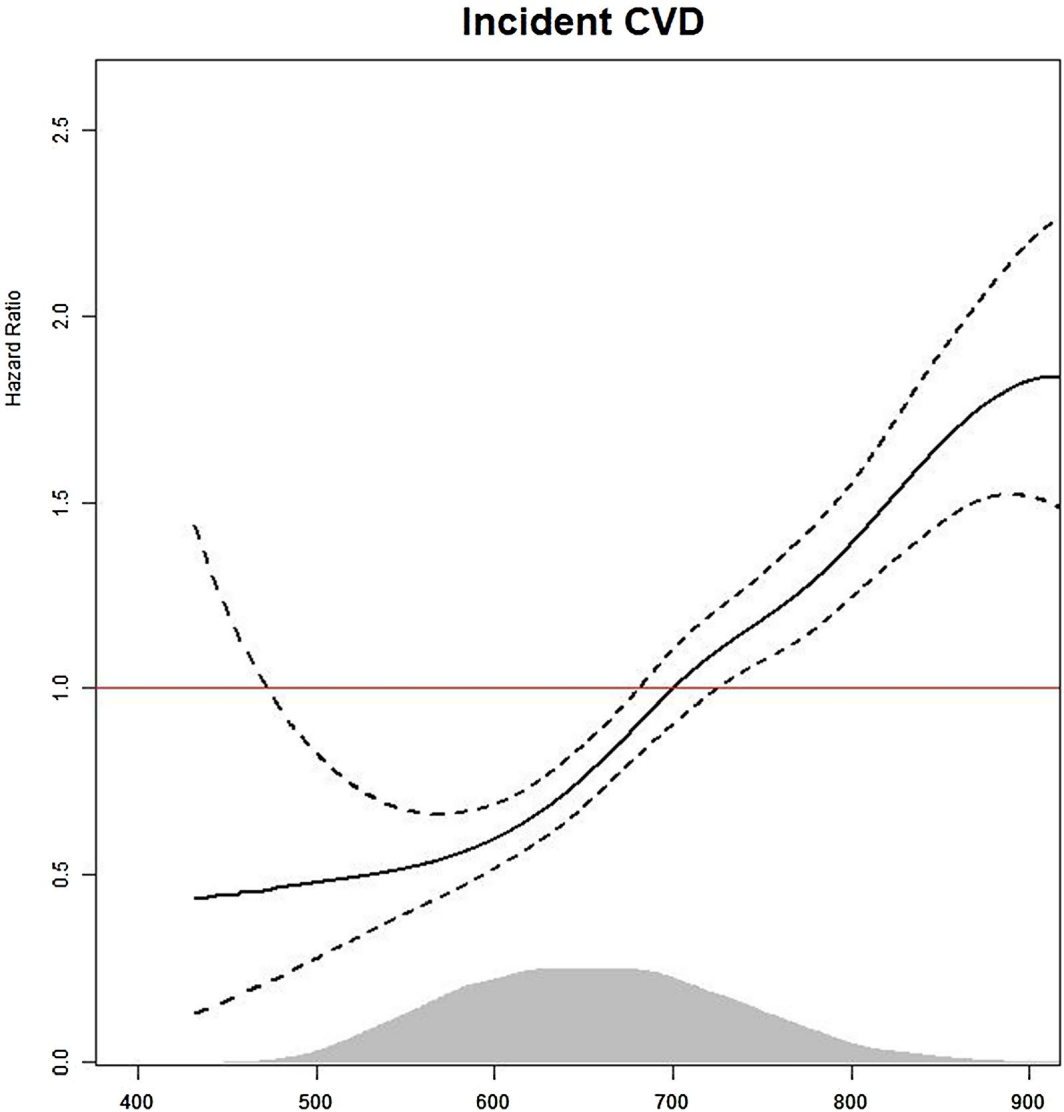
Cox proportional hazard spline curve for incident CVD and density plot according to TyG-WC index. The red line indicates a hazard ratio of 1. The black line represents the hazard ratio for incident CVD according to TyG-WC index. The dashed black area represents 95% confidence interval for hazard ratio. The grey area represents density of values. Abbreviations: CVD, cardiovascular disease

During the median 15.94 year of follow-up period, a total of 691 new-onset CVD cases were identified, thus representing 9.24% of the study population. The incidence rate of CVD per 1000 person-years was notably higher in the highest quartile (Q4 at 8.18; 7.14–9.22) as compared to the lowest quartile (Q1 at 3.12; 2.49–3.75). Figure 2 illustrates the Kaplan–Meier curves displaying the cumulative incidence rates of CVD and all-cause mortality in relation to the TyG-WC index quartiles. During the follow-up period, the cumulative incidence rate of CVD was significantly higher in the groups with higher TyG-WC index quartiles than in those with lower quartiles, as determined by the log-rank test (*P* < 0.001). However, the cumulative incidence of all-cause mortality did not exhibit a consistent pattern with increasing TyG WC index quartiles. Cox proportional hazard spline curves illustrated that the TyG-WC index exhibited a dose-dependent positive correlation with incident CVD, as depicted in Fig. 3. Table 2 presents the results of the modified Cox regression analysis by Fine and Gray method, investigating the association between quartiles of the TyG-WC index and incident CVD, with all-cause mortality as a competing risk. In the univariate model, the higher TyG-WC index group showed a significantly higher HR (95% CI) for CVD incidence than the lower quartile group. This significant relationship persisted across all the adjusted models. In Model 3, after accounting for all-cause mortality as a competing risk, the HR and 95% CI for the incidence of CVD among the quartiles ranged from 1.47 (1.12–1.93), 1.91 (1.44–2.54) and 2.24 (1.63–3.07) in Q2–Q4, respectively. However, the relationship between TyG-WC index quartiles and all-cause mortality showed no statistically significant results in any of the models when considering CVD as a competing risk. Within the examined CVD subtypes, angina and stroke were significantly associated with the TyG-WC index, with HR (95% CI) of 2.43 (1.49–3.98) for angina and 1.75 (1.13–2.70) for stroke in the Q4, as detailed in Additional file 2: Table S1. In the case of myocardial infarction, a significant association was noted up to Model 1 with a HR (95% CI) of 2.72 (1.12–6.57) in Q4. However, this correlation diminished in the analyses of subsequent models.

**Table 2:** Competing risk regression model of CVD and all-cause mortality based on the TyG-WC index quartiles TyG-WC index quartiles

	TyG-WC index quartiles									
	Q1		Q2			Q3			Q4	
	(*n* = 1872)		(*n* = 1869)			(*n* = 1870)			(*n* = 1871)	
Incident CVD, n	95		157			200			239	
Follow-up, person-year	30474.5		29969.4			29610.3			29209.3	
Incident rate per 1000 person-year (95% CI)	3.12 (2.49–3.75)		5.24 (4.42–6.06)			6.75 (5.81–7.69)			8.18 (7.14–9.22)	

	HR	HR	95% CI	***P*** value	HR	95% CI	***P*** value	HR	95% CI	***P*** value
Risk of incident CVD										
Unadjusted	1 (ref.)	1.68	1.30–2.16	< 0.001	2.17	1.70–2.77	< 0.001	2.65	2.09–3.36	< 0.001
Model 1	1 (ref.)	1.55	1.20–2.02	< 0.001	2.04	1.55–2.67	< 0.001	2.46	1.83–3.30	< 0.001
Model 2	1 (ref.)	1.53	1.16–2.01	< 0.001	2.06	1.55–2.73	< 0.001	2.57	1.89–3.50	< 0.001
Model 3	1 (ref.)	1.47	1.12–1.93	< 0.001	1.91	1.44–2.54	< 0.001	2.24	1.63–3.07	< 0.001
All-cause death, n	116		129			138			179	
Follow-up, person-year	30474.5		29969.4			29610.3			29209.3	
Incident rate per 1000 person-year (95% CI)	3.81(3.12-4.50)		4.30(3.56–5.04)			4.66(3.88–5.23)			6.13(5.23–7.03)	

	HR	HR	95% CI	***P*** value	HR	95% CI	***P*** value	HR	95% CI	***P*** value
Risk of all-cause mortality										
Unadjusted	1 (ref.)	1.07	0.84–1.37	0.590	1.16	0.91–1.48	0.240	1.46	1.16–1.84	0.001
Model 1	1 (ref.)	0.92	0.71–1.21	0.550	1.04	0.78–1.39	0.790	1.37	0.98–1.92	0.060
Model 2	1 (ref.)	0.90	0.67–1.19	0.460	1.04	0.76–1.43	0.800	1.30	0.90–1.86	0.160
Model 3	1 (ref.)	0.86	0.65–1.15	0.310	0.97	0.71–1.33	0.850	1.11	0.76–1.61	0.590

Model 1: adjusted for sex, age and body mass index

Model 2: adjusted for variables used in Model 1 plus residence, total energy intake, smoking status, drinking status and physical activity

Model 3: adjusted for variables used in Model 2 plus mean blood pressure, fasting plasma glucose, serum total cholesterol, CRP and eGFR

CVD, cardiovascular disease; HR, hazard ratio; CRP, C-reactive protein; eGFR, estimated glomerular filtration rate

### Subgroup analyses

Additional file 2: Table S2 presents the results of subgroup analysis stratified by age, sex, obesity status, HTN, DM and dyslipidemia. For the age subgroups, the HR (95% CI) for Q4 compared with the referent Q1 were as follows: 2.77 (1.48–5.17) for those in their 40 s, 2.38 (1.44–3.96) for those in their 50 s, and 2.30 (1.39–3.81) for those in their 60 s. Similarly, regarding sex, the HR (95% CI) for Q4 were 2.06 (1.33–3.18) for men and 2.79 (1.70–4.57) for women. Regarding obesity status, both obese and non-obese groups exhibited a significant increase in CVD risk within Q4, with HR (95% CI) of 1.96 (1.32–2.91) for the obese group and 1.62 (1.14–2.32) for the non-obese group, respectively. In the medical history, HTN was associated with HR (95% CI) of 2.49 (1.56–4.00) in Q4 and 2.54 (95% CI 1.65–3.89) for those absence of HTN. For dyslipidemia, the HR (95% CI) in Q4 were 2.12 (1.36–3.29) for those with the condition and 1.88 (1.20–2.95) for those without. In the subgroup without DM, the HR (95% CI) in Q4 was 2.56 (1.83–3.58), whereas for patients with DM, the HR (95% CI) was observed to be 2.00 (0.95–4.18). Additionally, there were no significant associations between the TyG-WC index and all-cause mortality across all stratified age groups, sexes, obesity statuses and medical history.

## Discussion

We verified the relationship between the TyG-WC index and the occurrence of CVD even when considering all-cause mortality as a competing risk. These findings imply that monitoring the TyG-WC index could serve as a valuable aid in evaluating the risk of CVD. Furthermore, we suggest that the risk of CVD increased in a dose-dependent manner as the TyG-WC index increased.

Our results were consistent with previous studies, thereby suggesting that the TyG-WC index may serve as an indicator that explains the occurrence of CVD [21, 22, 32].

A previous cross-sectional study of Korean adults suggested that the TyG-WC index reflected subclinical atherosclerosis as measured by coronary artery calcification and demonstrated the highest predictive value compared to TyG index, TyG-BMI index, and HOMA-IR [21]. Moreover, a cohort study of Chinese with HTN and obstructive sleep apnea revealed that higher baseline TyG-WC levels were associated with an increased risk of myocardial infarction [22]. This suggests that the TyG-WC index may be a valuable tool for identifying additional CVD risks. In our study, we observed a consistent trend indicating an increased incidence of CVD events with elevated TyG-WC indices. Furthermore, we extended our research to encompass the general population, rather than limiting it to specific disease groups. Thus, our investigation differentiated itself by considering a broad range of CVD events. Instead of confining the analysis to subclinical atherosclerosis or isolated myocardial infarction, we included myocardial infarction, angina pectoris, peripheral artery disease, and stroke to provide a more comprehensive perspective.

Recent studies have established an association between the TyG index and CVD, thereby highlighting its reliability as an indicator of IR [11, 18, 20, 33]. IR affects various tissues, influences the reduction of glucose transporter type 4 within skeletal muscles, and impairs glycogen synthesis in the liver, while increasing gluconeogenesis as well as involving the mutual interaction and involvement of free fatty acids [34, 35]. During its development, there is a concurrent impairment in the metabolism of fatty acids, which can influence their movement and accumulation from adipose to non-adipose tissues, such as skeletal muscles or liver tissue [36, 37].

Central obesity is a better predictor of heightened DM and CVD risk than general obesity; it more accurately reflects chronic inflammation and IR [38–40]. Several studies have indicated that the negative metabolic effects of excess fat were more strongly associated with its distribution rather than its quantity [41, 42]. As an indicator of fat distribution, WC has been utilized as an effective marker for central obesity in various risk scores and definitions of metabolic syndrome, and has been shown to be strongly correlated with HTN and DM [43–45].

In this study, we observed that each TyG-WC index quartile showed an increasing trend in both the TyG index and WC as the quartile increased. The increase in both the TyG index and WC values was well-documented to be associated with CVD risk elevation from the perspective of increased IR and central obesity. A potential explanation was that the ultimate outcome was an increase in CVD risk when these two metrics, which comprise the TyG-WC index, increased concurrently.

In the subtype-specific analysis of CVD, significant associations with the TyG-WC index were found for angina and stroke, given adequate sample sizes. For myocardial infarction, we believe that a significant association with the TyG-WC index and incident myocardial infarction could have been observed in a larger population. Moreover, the analysis of peripheral artery disease was inconclusive due to insufficient sample size. Therefore, follow-up studies using large population groups are necessary. Subgroup analysis by age, sex, obesity, HTN and dyslipidemia demonstrated that higher TyG-WC index quartiles consistently indicate an increased risk of CVD, even after adjusting for confounders and considering death as a competing risk. This underscores the potential of TyG-WC index as a universal predictor of CVD risk in the general population. For the subgroup with DM, the presence of DM in only 779 participants (10.4%) might have limited the ability to establish a significant association between the TyG-WC index and incident CVD. Additionally, the prevalent prescription of metformin as a first-line treatment in Korea, due to its insulin-sensitizing properties, may have lessened the observed effects in our analysis.

It is worth noting that when considering CVD as a competing risk, the TyG-WC index did not exhibit statistical significance in relation to all-cause mortality. Although there was some partial statistical significance in the highest TyG index quartiles in the univariate model, and HRs generally increased as quartiles rose across all models, the statistical significance remained insufficient. These findings suggest that confounding factors, in addition to the components of the TyG-WC index such as IR and central obesity, may be associated with all-cause mortality, thus making it challenging to establish a direct causal relationship.

This study had several limitations. First, although we employed well-trained examiners and conducted in-depth face-to-face interviews to validate each case, it is essential to acknowledge the possibility of information bias when characterizing CVD events because of our reliance on self-reported questionnaires. Nonetheless, the reliability of our approach was reinforced by a prior study that discovered a significant 93% agreement between self-reported diagnoses and those confirmed through physician evaluations of medical records [46]. Secondly, we did not account for potential changes in medication use during the follow-up period, such as lipid-lowering, anti-hypertensive, and anti-diabetic medications, which may have affected the outcomes of interest. Third, markers that are speculated to be associated with possible insulin resistance, such as uric acid, were not included in the data of this study, making it impossible to reflect on them in detail [47]. Fourth, this study specifically targeted the Korean population based on prior studies indicating the strong explanatory potential of the TyG-WC index for IR, especially in individuals of Asian ethnicity [15, 16]. Therefore, the statistical explanatory power of the TyG-WC index may vary across racial groups. Finally, we could not establish a causal relationship between TyG-WC index and CVD. Additional research involving large-scaled clinical trials or Mendelian randomization studies may be needed to establish their causal relationship. Despite these limitations, this study demonstrated a significant association between the TyG-WC index and the occurrence of CVD, even when all-cause mortality was considered as a competing risk using competing risk regression.

In conclusion, this study suggests that the TyG-WC index can be a useful indicator to predict the risk of CVD. Furthermore, our results suggest the need for further research to explore its utility in greater depth.

### Supplementary Information

**Additional file 1: Figure S1.** Log–log plot of the risk of incident CVD by the TyG-WC index quartile.

**Additional file 2: Table S1.** Competing risk regression model of CVD categorized by subtypes based on the TyG-WC index quartiles. **Table S2**. Subgroup regression analysis model examining the relationship between CVD and the TyG-WC index, categorized by age, with all-cause mortality as a competing risk.

## Data Availability

The dataset used in this study was obtained by reviewing and evaluating previous research. Plan of Korea Disease Control and Prevention Agency (https://www.kdca.go.kr).

## Abbreviations

CVD: Cardiovascular disease
IR: Insulin resistance
HOMA-IR: Homeostatic assessment of insulin resistance
TyG: Triglyceride glucose
WC: Waist circumference
BMI: Body mass index
KoGES: Korean Genome and Epidemiology Study
KDCA: Korea Disease Control and Prevention Agency
MET: Metabolic equivalent of task
KRW: Korean Won
HRs: Hazard ratios
CRP: C-reactive protein
DM: Diabetes mellitus
HTN: Hypertension
eGFR: Estimated glomerular filtration rate
FPG: Fasting plasma glucose
MBP: Mean blood pressure
