# Supplementary material for: Triglyceride-glucose-waist circumference index predicts the incidence of cardiovascular disease in Korean populations: competing risk analysis of an 18-year prospective study
Source: Eur J Med Res. 2024 Apr 2;29:214. doi: 10.1186/s40001-024-01820-9 (PMC10985901; doi:10.1186/s40001-024-01820-9)
Supplement: Supplementary file 1 — Additional file 1: Figure S1. Log–log plot of the risk of incident CVD by the TyG-WC index quartile. [file 40001_2024_1820_MOESM1_ESM.docx]

**Figure S1.** Log-log plot of the risk of incident CVD by the TyG-WC index quartile.

**
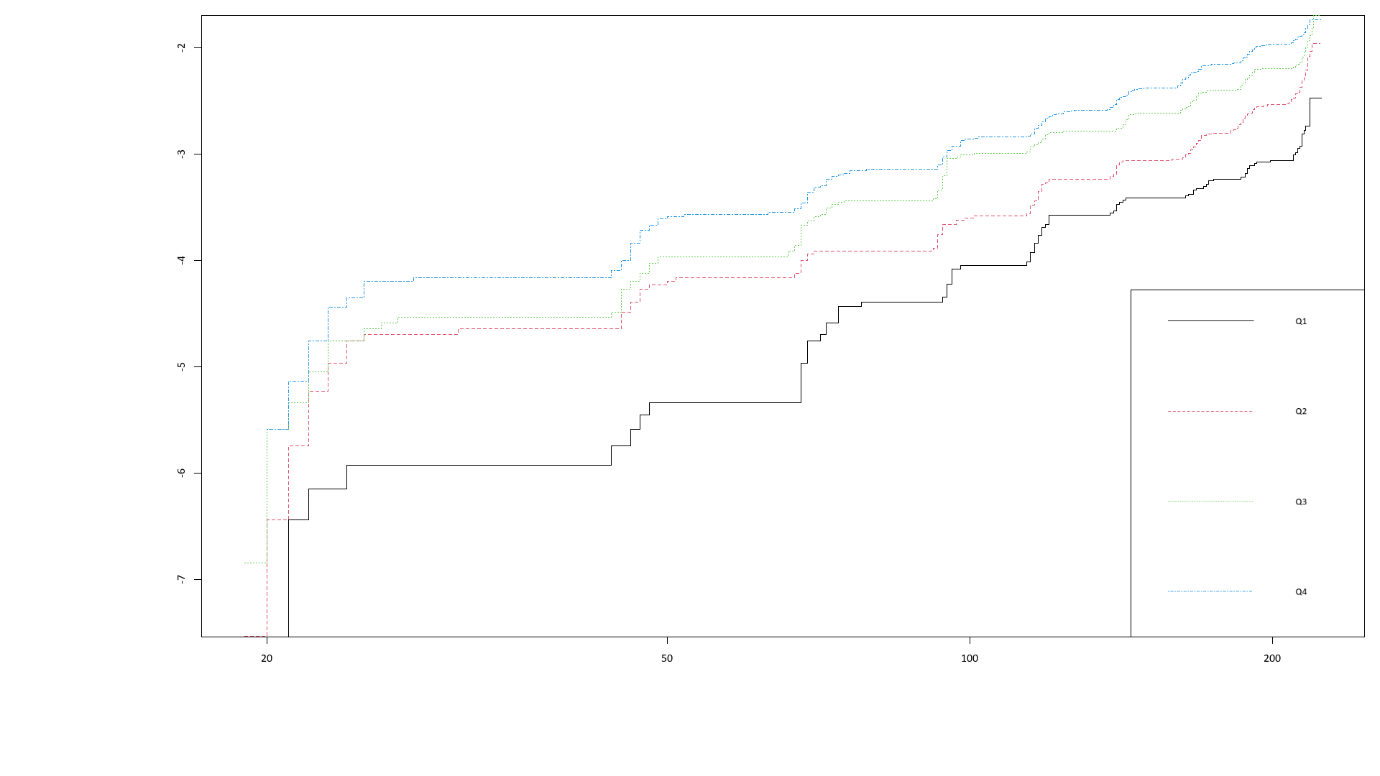
**

CVD, cardiovascular disesae; TyG-WC; triglyceride-glucose-waist circumference.
