# Supplementary material for: Triglyceride-glucose-waist circumference index predicts the incidence of cardiovascular disease in Korean populations: competing risk analysis of an 18-year prospective study
Source: Eur J Med Res. 2024 Apr 2;29:214. doi: 10.1186/s40001-024-01820-9 (PMC10985901; doi:10.1186/s40001-024-01820-9)
Supplement: Supplementary file 2 — Additional file 2: Table S1. Competing risk regression model of CVD categorized by subtypes based on the TyG-WC index quartiles. Table S2. Subgroup regression analysis model examining the relationship between CVD and the TyG-WC index, categorized by age, with all-cause mortality as a competing risk. [file 40001_2024_1820_MOESM2_ESM.docx]

**Table S1.** Competing risk regression model of CVD categorized by subtypes based on the TyG-WC index quartiles.

|  | TyG-WC index quartiles | | | | | | | | | | | | | | | | | | |
| --- | --- | --- | --- | --- | --- | --- | --- | --- | --- | --- | --- | --- | --- | --- | --- | --- | --- | --- | --- |
|  | Q1 |  | | Q2 | |  | |  | | | Q3 |  | |  | | | Q4 |  | |
|  | (*n* = 1872) | |  | | (*n* = 1869) | |  | |  | (*n* = 1870) | | |  | |  | (*n* = 1871) | | |  |
| Incident MI, n | 10 | |  | | 25 | |  | |  | 30 | | |  | |  | 45 | | |  |
| Follow-up, person-year | 27417.9 | |  | | 27314.9 | |  | |  | 26981.7 | | |  | |  | 27056.6 | | |  |
| Incident rate per  1000 person-year (95% CI) | 0.36 (0.13–0.59) | | | | 0.92 (0.56–1.28) | | | |  | 1.11 (0.71–1.51) | | | | |  | 1.66 (1.17–2.15) | | | |
|  | HR | | HR | | 95% CI | | *P* value | | HR | 95% CI | | | P value | | HR | 95% CI | | | *P* value |
| Risk of incident MI |  | |  | |  | |  | |  |  | | |  | |  |  | | |  |
| Unadjusted | 1 (ref) | | 2.49 | | 1.2–5.19 | | 0.015 | | 3.07 | 1.5–6.27 | | | 0.002 | | 4.56 | 2.3–9.03 | | | <0.001 |
| Model 1 | 1 (ref) | | 1.95 | | 0.90–4.23 | | 0.089 | | 2.11 | 0.95–4.71 | | | 0.068 | | 2.72 | 1.12–6.57 | | | 0.027 |
| Model 2 | 1 (ref) | | 1.95 | | 0.89–4.27 | | 0.095 | | 1.78 | 0.77–4.13 | | | 0.180 | | 2.50 | 1.00–6.29 | | | 0.051 |
| Model 3 | 1 (ref) | | 1.78 | | 0.81–3.88 | | 0.150 | | 1.49 | 0.64–3.43 | | | 0.350 | | 1.76 | 0.69–4.49 | | | 0.240 |
| Incident angina, n | 35 | |  | | 68 | |  | |  | 82 | | |  | |  | 86 | | |  |
| Follow-up, person-year | 27259.1 | |  | | 27030.3 | |  | |  | 26723.9 | | |  | |  | 26822.1 | | |  |
| Incident rate per  1000 person-year (95% CI) | 1.28 (0.85–1.71) | | | | 2.52 (1.92–3.12) | | | |  | 3.07 (2.41–3.73) | | | | |  | 3.21 (2.53–3.89) | | | |
|  | HR | | HR | | 95% CI | | *P* value | | HR | 95% CI | | | *P* value | | HR | 95% CI | | | *P* value |
| Risk of incident angina |  | |  | |  | |  | |  |  | | |  | |  |  | | |  |
| Unadjusted | 1(ref) | | 1.96 | | 1.30–2.94 | | 0.001 | | 2.40 | 1.62–3.56 | | | <0.001 | | 2.49 | 1.68–3.69 | | | <0.001 |
| Model 1 | 1(ref) | | 1.88 | | 1.25–2.83 | | 0.003 | | 2.35 | 1.54–3.59 | | | <0.001 | | 2.46 | 1.55–3.89 | | | <0.001 |
| Model 2 | 1(ref) | | 1.82 | | 1.19–2.79 | | 0.006 | | 2.37 | 1.52–3.68 | | | <0.001 | | 2.55 | 1.58–4.12 | | | <0.001 |
| Model 3 | 1(ref) | | 1.80 | | 1.17–2.75 | | 0.007 | | 2.32 | 1.49–3.60 | | | <0.001 | | 2.43 | 1.49–3.98 | | | <0.001 |
| Incident PAD, n | 3 | |  | | 4 | |  | |  | 3 | | |  | |  | 5 | | |  |
| Follow-up, person-year | 27433.9 | |  | | 27304.5 | |  | |  | 27134.7 | | |  | |  | 27293.9 | | |  |
| Incident rate per  1000 person-year (95% CI) | 0.11 (0.0–0.23) | | | | 0.15 (0.01–0.29) | | | |  | 0.11 (0.00–0.24) | | | | |  | 0.18 (0.02–0.34) | | | |
|  | HR | | HR | | 95% CI | | *P* value | | HR | 95% CI | | | *P* value | | HR | 95% CI | | | *P* value |
| Risk of incident PAD |  | |  | |  | |  | |  |  | | |  | |  |  | | |  |
| Unadjusted | 1(ref) | | 1.33 | | 0.30–5.92 | | 0.710 | | 1.01 | 0.20–4.97 | | | 0.990 | | 1.67 | 0.40–6.96 | | | 0.480 |
| Model 1 | 1(ref) | | 0.83 | | 0.26–5.47 | | 0.810 | | 0.83 | 0.12–5.79 | | | 0.850 | | 1.13 | 0.20–6.53 | | | 0.890 |
| Model 2 | 1(ref) | |  | | *N/A* | |  | |  | *N/A* | | |  | |  | *N/A* | | |  |
| Model 3 | 1(ref) | |  | | *N/A* | |  | |  | *N/A* | | |  | |  | *N/A* | | |  |
| Incident stroke, n | 58 | |  | | 64 | |  | |  | 95 | | |  | |  | 118 | | |  |
| Follow-up, person-year | 27258.7 | |  | | 27217.9 | |  | |  | 26772.0 | | |  | |  | 26849.6 | | |  |
| Incident rate per  1000 person-year (95% CI) | 2.13 (1.58–2.68) | | | | 2.35 (1.77–2.93) | | | |  | 3.55 (2.84–4.26) | | | | |  | 4.39 (3.6–5.18) | | | |
|  | HR | | HR | | 95% CI | | *P* value | | HR | 95% CI | | | *P* value | | HR | 95% CI | | | *P* value |
| Risk of incident stroke |  | |  | |  | |  | |  |  | | |  | |  |  | | |  |
| Unadjusted | 1(ref) | | 1.10 | | 0.77–1.57 | | 0.600 | | 1.68 | 1.21–2.33 | | | 0.002 | | 2.07 | 1.51–2.84 | | | <0.001 |
| Model 1 | 1(ref) | | 1.00 | | 0.70–1.44 | | 0.980 | | 1.55 | 1.07–2.24 | | | 0.022 | | 1.92 | 1.27–2.90 | | | 0.002 |
| Model 2 | 1(ref) | | 1.06 | | 0.73–1.55 | | 0.760 | | 1.68 | 1.13–2.48 | | | 0.010 | | 2.04 | 1.32–3.14 | | | 0.001 |
| Model 3 | 1(ref) | | 1.02 | | 0.69–1.48 | | 0.940 | | 1.55 | 1.04–2.30 | | | 0.030 | | 1.75 | 1.13–2.70 | | | 0.012 |

Model 1: adjusted for sex, age and body mass index.

Model 2: adjusted for variables used in Model 1 plus residence, total energy intake, smoking status, drinking status and physical activity.

Model 3: adjusted for variables used in Model 2 plus mean blood pressure, fasting plasma glucose, serum total cholesterol, CRP and eGFR.

Abbreviations: CVD, cardiovascular disease; HR, hazard ratio; CRP, C-reactive protein; eGFR, estimated glomerular filtration rate; MI, myocardial infarction; PAOD, peripheral artery disease.

**Table S2**. Subgroup regression analysis model examining the relationship between CVD and the TyG-WC index, categorized by age, with all-cause mortality as a competing risk.

|  | TyG-WC index quartiles | | | | | | | | | |  |
| --- | --- | --- | --- | --- | --- | --- | --- | --- | --- | --- | --- |
|  | Q1 |  | Q2 |  |  | Q3 |  |  | Q4 |  |  |
| Risk of incident CVD | HR | AHR^*^ | 95% CI | *P* value | AHR | 95% CI | *P* value | AHR | 95% CI | *P* value |  |
| Sex |  |  |  |  |  |  |  |  |  |  |  |
| Men | 1 (ref) | 1.38 | 0.98–1.95 | 0.070 | 1.27 | 0.85–1.89 | 0.250 | 2.06 | 1.33–3.18 | 0.001 |  |
| Women | 1 (ref) | 1.64 | 1.08–2.48 | 0.020 | 2.11 | 1.36–3.26 | <0.001 | 2.79 | 1.70–4.57 | <0.001 |  |
| Age groups |  |  |  |  |  |  |  |  |  |  |  |
| 40s | 1 (ref) | 1.63 | 0.98–2.70 | 0.061 | 2.11 | 1.22–3.66 | 0.008 | 2.77 | 1.48–5.17 | 0.001 |  |
| 50s | 1 (ref) | 1.51 | 0.99–2.31 | 0.060 | 1.87 | 1.18–2.96 | 0.008 | 2.38 | 1.44–3.96 | <0.001 |  |
| 60s | 1 (ref) | 1.39 | 0.91–2.13 | 0.130 | 1.39 | 0.87–2.22 | 0.170 | 2.30 | 1.39–3.81 | 0.001 |  |
| Obesity status |  |  |  |  |  |  |  |  |  |  |  |
| Obese | 1 (ref) | 1.39 | 0.94–2.07 | 0.100 | 1.67 | 1.14–2.46 | 0.009 | 1.96 | 1.32–2.91 | <0.001 |  |
| Normal weight | 1 (ref) | 1.07 | 0.74–1.55 | 0.720 | 1.40 | 0.98–1.99 | 0.066 | 1.62 | 1.14–2.32 | 0.008 |  |
| Hypertension |  |  |  |  |  |  |  |  |  |  |  |
| presence | 1 (ref) | 1.82 | 1.24–2.66 | 0.002 | 1.73 | 1.13–2.64 | 0.012 | 2.49 | 1.56–4.00 | <0.001 |  |
| absence | 1 (ref) | 1.42 | 0.97–2.06 | 0.071 | 1.88 | 1.27–2.77 | 0.002 | 2.54 | 1.65–3.89 | <0.001 |  |
| DM | |  |  |  |  |  |  |  |  |  |  |
| presence | 1 (ref) | 0.87 | 0.46 1.66 | 0.670 | 1.78 | 0.95 3.33 | 0.072 | 2.00 | 0.95 4.18 | 0.067 |  |
| absence | 1 (ref) | 1.55 | 1.15 2.08 | 0.004 | 2.04 | 1.50 2.78 | <0.001 | 2.56 | 1.83 3.58 | <0.001 |  |
| Dyslipidemia |  |  |  |  |  |  |  |  |  |  |  |
| presence | 1 (ref) | 1.59 | 1.11–2.29 | 0.012 | 1.67 | 1.13–2.45 | 0.010 | 2.12 | 1.36–3.29 | <0.001 |  |
| absence | 1 (ref) | 1.10 | 0.74 –1.63 | 0.640 | 1.56 | 1.07–2.29 | 0.022 | 1.88 | 1.20–2.95 | 0.006 |  |
| Risk of all-cause mortality | HR | AHR^*^ | 95% CI | *P* value | AHR | 95% CI | *P* value | AHR | 95% CI | *P* value |  |
| Sex |  |  |  |  |  |  |  |  |  |  |  |
| Men | 1 (ref) | 0.62 | 0.43–0.91 | 0.013 | 0.96 | 0.64–1.45 | 0.850 | 0.94 | 0.56–1.60 | 0.830 |  |
| Women | 1 (ref) | 1.54 | 0.89–2.64 | 0.120 | 1.30 | 0.72–2.33 | 0.390 | 1.42 | 0.73–2.75 | 0.300 |  |
| Age groups |  |  |  |  |  |  |  |  |  |  |  |
| 40s | 1 (ref) | 1.40 | 0.70–2.80 | 0.340 | 1.31 | 0.60–2.89 | 0.500 | 1.20 | 0.46–3.18 | 0.710 |  |
| 50s | 1 (ref) | 0.76 | 0.39–1.45 | 0.400 | 1.03 | 0.53–1.99 | 0.940 | 0.93 | 0.42–2.08 | 0.860 |  |
| 60s | 1 (ref) | 0.85 | 0.69–1.24 | 0.390 | 1.28 | 0.86–1.89 | 0.220 | 1.43 | 0.90–2.27 | 0.130 |  |
| Obesity status |  |  |  |  |  |  |  |  |  |  |  |
| Obese | 1 (ref) | 1.83 | 1.09–3.08 | 0.023 | 1.77 | 1.06–2.96 | 0.029 | 1.53 | 0.89–2.64 | 0.120 |  |
| Normal weight | 1 (ref) | 0.82 | 0.59–1.16 | 0.260 | 0.75 | 0.53–1.06 | 0.110 | 0.86 | 0.61–1.21 | 0.380 |  |
| Hypertension | |  |  |  |  |  |  |  |  |  |  |
| presence | | 1 (ref) | 0.77 | 0.51–1.15 | 0.200 | 1.09 | 0.73–.1.63 | 0.680 | 1.00 | 0.63–1.57 | 0.990 |
| absence | | 1 (ref) | 1.18 | 0.81–1.73 | 0.390 | 0.89 | 0.58–1.35 | 0.580 | 1.12 | 0.65–1.93 | 0.690 |
| DM | |  |  |  |  |  |  |  |  |  |  |
| presence | | 1 (ref) | 1.49 | 0.79–2.81 | 0.220 | 1.54 | 0.78–3.05 | 0.210 | 1.37 | 0.61–3.06 | 0.450 |
| absence | | 1 (ref) | 0.84 | 0.62–1.15 | 0.280 | 0.77 | 0.54–1.08 | 0.130 | 0.98 | 0.67–1.44 | 0.920 |
| Dyslipidemia | |  |  |  |  |  |  |  |  |  |  |
| presence | | 1 (ref) | 0.78 | 0.50–1.22 | 0.280 | 1.15 | 0.74–1.79 | 0.540 | 1.02 | 0.60–1.73 | 0.940 |
| absence | | 1 (ref) | 1.00 | 0.69–1.47 | 0.980 | 0.66 | 0.42–1.04 | 0.078 | 1.00 | 0.59–1.70 | 0.990 |

Abbreviations: CVD, cardiovascular disease; HR, hazard ratio; AHR, adjusted hazard ratio; CRP, C-reactive protein; eGFR, estimated glomerular filtration rate; DM, diabetes mellitus.

^*^adjusted for variables used in sex, age, body mass, residence, total energy intake, smoking status, drinking status, physical activity, mean blood pressure, fasting plasma glucose, serum total cholesterol , CRP and eGFR.

Sex, age, body mass index, mean blood pressure, fasting plasma glucose and serum total cholesterol levels were excluded from the subgroup analysis for each component.
